# Decisional conflict, caregiver mastery, and depression among Chinese parental caregivers of children with leukemia

**DOI:** 10.1186/s12888-023-05084-1

**Published:** 2023-08-28

**Authors:** Mowen Liu, Weizhou Tang, Ye Zhang, Wenjun Sun, Yang Wang

**Affiliations:** 1 Department of Social Work and Social Policy, Renmin University of China, Beijing, China; 2 Beijing Yizhuang Technology Innovation Company Limited, Beijing, China; 3 Charles R. Drew University of Medicine and Science, Los Angeles, United States; 4 the High School Affiliated to Renmin University of China, Beijing, China; 5 Tianjin Di Ai Zhi Jia Hard-pressed Families Service Center, Tianjin, China

**Keywords:** Decisional conflict, Mastery, Depressive symptoms, Parental caregivers

## Abstract

**Background:**

Informal caregivers of children with leukemia can be emotionally and psychiatrically vulnerable when facing difficult treatment decisions (e.g., chemotherapy, targeted therapy, radiation, transplantation). A common behavioral manifestation of decisional conflict is the verbalized expression of uncertainty about which medical treatment plan to take. The study aims to examine the associations between decisional conflict, mastery, and depressive symptoms among parental caregivers of children with leukemia in China. It explored the mediating role of mastery in the relationship.

**Methods:**

A cross-sectional survey design was adopted. A total of 386 parental caregivers were recruited, and 325 valid questionnaires remained. The mean age of caregivers was 37.7 years, and 61.5% caregivers were female. We used Question Format Decisional Conflict Scale to assess decisional conflict, Pearlin’s Mastery Scale to assess mastery, and Center for Epidemiological Studies Depression 10 to assess depressive symptoms. We used mediation analyses to test the mediating effect of mastery.

**Results:**

The total score of decisional conflict scale, along with its dimensions of uncertainty, support, and effective decision were found negatively associated with depressive symptoms. In contrast, the dimension of information and value were not significantly associated with depressive symptoms. Mediation analyses demonstrated the direct effects of overall decisional conflict and uncertainly were fully mediated by mastery, while the direct effect of support and effective decision were partially mediated.

**Conclusions:**

Efforts should be made to alleviate parental caregivers’ decisional conflict and enhance sense of mastery. Particular attention should be paid to the psycho-social support to relieve uncertainties and ineffectiveness in decision making.

## Introduction

Leukemia is the most prevalent childhood cancer, constituting 28% of all pediatric cancers [1]. China had the highest age-standardized prevalence rate of leukemia in the world, with the greatest increase from 1990 to 2017 [2]. Each year, there are over 10,000 children with newly diagnosis of leukemia in China [3]. Despite advances in treatments and survival rates, leukemia still has substantial negative impacts on the mental well-being and quality of life of patients and families, especially when families face difficult treatment decisions (e.g., informal caregivers of children with leukemia can be emotionally and psychiatrically vulnerable when facing difficult treatment decisions (e.g., chemotherapy, targeted therapy, radiation, transplantation). Informal caregivers of patients can be emotionally and psychiatrically vulnerable when taking on caring responsibilities at each treatment phase [4, 5]. It is common that children with leukemia relied on their parents to make treatment decisions, which can be complicated, challenging, and stressful to them [6–8]. Although the involvement of patients and caregivers in health care is highlighted, parents can be scorched by the experience of dealing with the medical complexity [9, 10]. In addition, prior research indicated that caregiver’s psychological well-being is associated with sense of mastery [11]. The current study examined the role of mastery in the relationship between decisional conflict (DC) and depressive symptoms among parents of children with leukemia. It also examined potential variation of mastery’s role by dimension of DC.

Mastery is a construct that one perceives their global sense of control over life, and it plays as an important role in intervening the process of stress in the context of caregiving [12]. Empirical evidence indicated that in managing the threat of illness, mastery is powerful in explaining depressive symptoms in caregivers [13–15]. Caregivers could feel more burdened and experience and depressive symptoms due to a lack of mastery or a feeling of uncontrollable about their life [16, 17].

Pearlin’s Stress Process Model (SPM) has been extensively used to explain the formation of caregivers’ emotional problems as outcomes of various stressors. Primary stressors (e.g., cognitive and behavioral problems) are directly caregiving-related and objective, and they have both direct and indirect effects on psychological outcomes [18]. Secondary stressors (e.g., low levels of mastery, self-esteem, optimism, etc.) arise as responses to primary stressors and are more subjective [19, 20]. For instance, mastery, a sense of control one can perceive over their life situation, is regarded as a predictor of self-efficacy and can reflect one’s ability to cope with and adapt to challenging situations [17, 21]. Empirical evidence shows that mastery can intervene to protect caregivers from the impact of stress brought by caregiving, and a higher level of mastery could be associated with less depression [16, 17].

A common behavioral manifestation of DC is the verbalized expression of uncertainty about choices (e.g., which medical treatment plan to take). Specifically, a greater level of DC can come along with the expression of feeling a lack of information (e.g., have no idea where to take chemotherapy or radiotherapy), unclear about personal values (e.g., not sure which benefits and risks of each treatment option matter most), unsupported (e.g., few people can be relied on to share caregiving responsibilities), and being worried about not having made an effective decision (e.g., not satisfied with the decision) [22]. For each of the five dimensions above, there are theories and models suggesting the associations between them and individuals’ psychological well-being. For example, theory of human relatedness asserts that the experience of social support and sense of belonging negatively predict depression among older adults [23]. Chiavari et al. (2015) developed a decisional support intervention aiming to provide young patients with assistance to get access to medical information, to clarify values, to identify social and material resources, and to form an eventual decision plan [24].

Recent studies mainly focused on the process of medical treatment decisions made by patients and parents of children, communications between parents, children, and oncologists, and effectiveness of decision support interventions [25–28]. However, to our knowledge, limited studies have examined the pathway between DC, mastery, and depression. Given the theoretical base and the context of parents of children with leukemia, it is rational for this study to hypothesize that mastery mediates the relationship between DC and depression, and the mediating effect can vary across five dimensions of DC. Insights from this study can help healthcare professionals develop deeper understanding about the effect of DC on depression through mastery, and assist them in alleviating depressive symptoms more effectively.

## Methods

### Study setting and participants

The current study used a cross-sectional design, collecting data from November 2021 to July 2022 through an instant messaging and social media mobile app (WeChat). Data were obtained using a self-report questionnaire. This study was approved by the Committee on Human Research Publication and Ethics, Renmin University of China (2,020,030,054).

Participants of this study were recruited at Di Ai Zhi Jia Hard-pressed Families Service Center, which is a non-profit organization initiated by medical professionals, former patients and their families, and has now developed into a professional medical social work service institution. All of the participants had received services from the center, and they were recruited via the online community built by the center. Participants completed the questionnaire after being informed about the content of the questionnaire and gave their informed consents. Eligible participants were parental caregivers of children with leukemia. Caregivers of patients older than 18 years old were excluded from this study. A total of 386 people completed the questionnaire, and finally 325 valid questionnaires remained.

### Measures

#### Demographic and caregiver-related questionnaire

Demographic variables included gender (male/ female), age, educational level (primary school and below/ junior high school or technical secondary school/ high school or college/ university or above), employment status (staff of the state/enterprises/institutions/ business/service/manufacturing workers/ freelancers/self-employed/ farmers/herdsmen/fishermen/ unemployed) and partner status (unmarried/ first marriage and living together/ separated but not divorced/ divorced/ remarried/ widowed). The patients’ medical characteristics included current treatment status (planned/active/chemotherapy/radiotherapy/finished) and length of time from diagnosis (in months). Except for the variables mentioned above, insurance reimbursement percentage, chronic diseases, smoking status (smoking every day/ smoking sometimes/ has given up smoking/ never smoking), and drinking status (never drinking/ has given up drinking/ no more than once a week/ 2–3 times a week/ 4 or more times a week) were also included as control variables. Among them, age, length of time from diagnosis, insurance reimbursement percentage and chronic diseases were continuous variables, and the rest were categorical variables.

#### Depressive symptoms

Depressive symptoms were measured using the Center for Epidemiological Studies Depression 10 Scale (CES-D-10) [29], which was a self-completed questionnaire that scores the severity of depressive symptoms in general populations. The scale consisted of 10 questions, each of which was divided into four levels from 1 (rarely or none of the time) to 4 (most or all of the time). The possible range of CES-D-10 score ranged from 10 to 40 (after reversing the positive items: “I was happy” and “I felt hopeful about the future). A higher total score indicated more depressive symptoms. The Cronbach’s α coefficient was 0.85 in this study.

#### Decisional conflict

DC was measured with Decisional Conflict Scale (DCS) [30]. This scale contained 16 items measuring personal perceptions of the following five domains: (a) uncertainty in choosing options; (b) feeling uninformed; (c) unclear about personal values; (d) feeling unsupported in decision making; and (e) effective decision making. Likert 5-point rating method was adopted for all questions. The total score ranged from 0 to 64. The calculation procedures of the total score and subscales were: (1) scores of each item were summed; (2) divided by 16; and (3) multiplied by 25. A higher score indicated a more serious DC. The Cronbach’s α coefficient was 0.92 in this study.

#### Mastery

Mastery was measured with Pearlin’s Mastery Scale [31]. The scale consisted of 7 questions. Likert 4-point rating method was adopted for all questions. Each item was scored from 1 (strongly agree) to 4 (strongly disagree). The total score ranged from 7 (lowest sense of mastery) to 28 (highest sense of mastery) after reversing the negative items. The possible range of total score was from 7 to 28. A higher total score indicates a higher level of mastery. The Cronbach’s α coefficient was 0.82 in this study.

### Data analysis

Statistical analyses were performed in Stata (version 17.0). First, for all variables of socio-demographic and clinical characteristics, descriptive statistics were presented as means and standard deviations (SD) or counts and percentages (%) for continuous variables and categorical variables, respectively. Second, correlation analyses were conducted to investigate the association between overall DCS and its subscales, and mastery and depressive symptoms. The p value less than 0.05 represented a significant correlation. Third, regression analyses were performed with overall DCS and its subscales as independent variables respectively, depressive symptoms as the dependent variable, and mastery as the only included mediator, controlling for the socio-demographic and clinical characteristics (e.g., chronic diseases, length of time from diagnosis, and current treatment stage). The indirect effect (a*b) was estimated as the product of regression coefficients, which predicted overall DC (a) and depressive symptoms from mastery (b). Bootstrapping techniques were used to reveal the significant indirect effects of overall DCS and its subscales on depressive symptoms through mastery, which was considered to be suitable for reliable estimation of small sample size. Based on the percentile bootstrapping method, unstandardized coefficients and their confidence intervals (CI) were estimated. The 95% confidence interval of indirect effect did not contain zero, indicating that there was a significant indirect effect. There were no missing data since the online survey required respondents to answer all questions.

## Results

### Participants

All of the participants provided written informed consent. Table 1 presents caregivers’ socio-demographic characteristics and health behaviors, as well as patients’ clinical information. The mean age of caregivers was 37.7 years (SD = 5.7, range 24–54), and most caregivers were female (61.5%). Less than half of the caregivers had a junior high school or technical secondary school education (49.9%). Around three quarters of the caregivers were unemployed at the time of completing the survey. Among the unemployed caregivers, 196 people’s unemployment occurred after their children fell ill, accounting for 80.0%. Of the 325 caregivers, 217 (66.8%) had their marital status as first marriage or living together. Almost half of the patients’ current treatment status was “active” (47.1%). The average length of time since diagnosis was 25.0 months (SD = 24.0, range 0-174). Nearly 80% of caregivers had no chronic diseases. Nearly two-thirds of the caregivers never smoked or drank.

**Table 1:** Demographic, patients’ clinical characteristics, and caregivers’ personal habits (N = 325)

	N(%) or mean(SD)
Gender (female)	200(61.5%)
Age (in years)	37.7(5.7)
Education level	
Primary school and below	45(13.2%)
Junior high school or technical secondary school	162(49.9%)
High school or college	68(20.9%)
University or above	52(16.0%)
Employment status	
Staff of the state/enterprises/institutions	17(5.2%)
Business/service/manufacturing workers	11(3.4%)
Freelancers/self-employed	22(6.8%)
Farmers/herdsmen/fishermen	30(9.2%)
Unemployed	245(75.4%)
Marital status	
Unmarried	8(2.5%)
First marriage and living together	217(66.8%)
Separated but not divorced	52(16.0%)
Divorced	24(7.4%)
Remarried	22(6.8%)
Widowed	2(0.6%)
Current treatment stage of children	
planned	15(4.6%)
active	153(47.1%)
chemotherapy	38(11.7%)
radiotherapy	2(0.6%)
finished	117(36.0%)
Length of time from diagnosis of children (in months)	25.0(24.0)
Insurance reimbursement percentage	39.5(14.1)
Chronic diseases	0.3(0.8)
Smoking status	
Smoking every day	34(10.5%)
Smoking sometimes	23(7.1%)
Has given up smoking	28(8.6%)
Never smoking	240(73.9%)
Drinking status	
Never drinking	224(68.9%)
Has given up drinking	52(16.0%)
No more than once a week	32(9.9%)
2–3 times a week	8(2.5%)
4 or more times a week	9(2.8%)
Overall decisional conflict	23.1(18.8)
Informed subscale	5.9(2.9)
Values Clarity subscale	5.7(2.7)
Support subscale	6.0(3.1)
Uncertainty subscale	6.8(2.7)
Effective decision subscale	6.4(3.2)
Mastery	16.5(3.2)
Depressive symptoms	24.8(5.8)

### Depressive symptoms, decisional conflict, and mastery

There are five subscales included in DC. The following description was drawn by comparing the mean scores of the five dimensions. The two lowest subscales of DC were informed decision-making subscale and values clarity subscale, with their mean scores 5.9 (SD = 2.9, range 3–15) and 5.7 (SD = 2.7, range 3–15) respectively. Decision-making uncertainty subscale had the highest mean score, with a score of 6.8 (SD = 2.7, range 3–15). The mean depressive symptoms and mastery scores were 24.8 (SD = 5.8, range 10–40) and 16.5 (SD = 3.2, range 7–28), respectively.

Bivariate correlation analysis results showed that overall DC was positively related to depressive symptoms (r = 0.16, p < 0.01). Regarding the relationship between subscale of DC and depressive symptoms, except for decisional-making informed subscale (r = 0.08, p > 0.05) and values clarity subscale (r = 0.05, p > 0.05), other subscales were significantly positively correlated with depressive symptoms (see Table 2). Mastery had significant negative correlation with all DC subscales except values clarity subscale. There was a significant negative correlation between mastery and depressive symptoms. Among all subscales, the decision-making uncertainty subscale had the strongest correlation with mastery.

**Table 2:** Descriptive results and correlations among overall decisional conflict and its subscales, mastery and depressive symptoms (N = 325)

	1	2	3	4	5	6	7	8
1. Depressive symptoms	1.000							
2. Mastery	-0.507**	1.000						
3. Overall decisional conflict	0.157**	-0.157**	1.000					
4. Informed subscale	0.083	-0.110*	0.819**	1.000				
5. Values Clarity subscale	0.047	-0.099	0.835**	0.735**	1.000			
6. Support subscale	0.177**	-0.140*	0.820**	0.531**	0.555**	1.000		
7. Uncertainty subscale	0.158**	-0.174**	0.783**	0.506**	0.534**	0.634**	1.000	
8. Effective decision subscale	0.170**	-0.121*	0.834**	0.593**	0.617**	0.612**	0.556**	1.000

*p < 0.05, **p < 0.01

### Mediation analyses

**Fig. 1:**
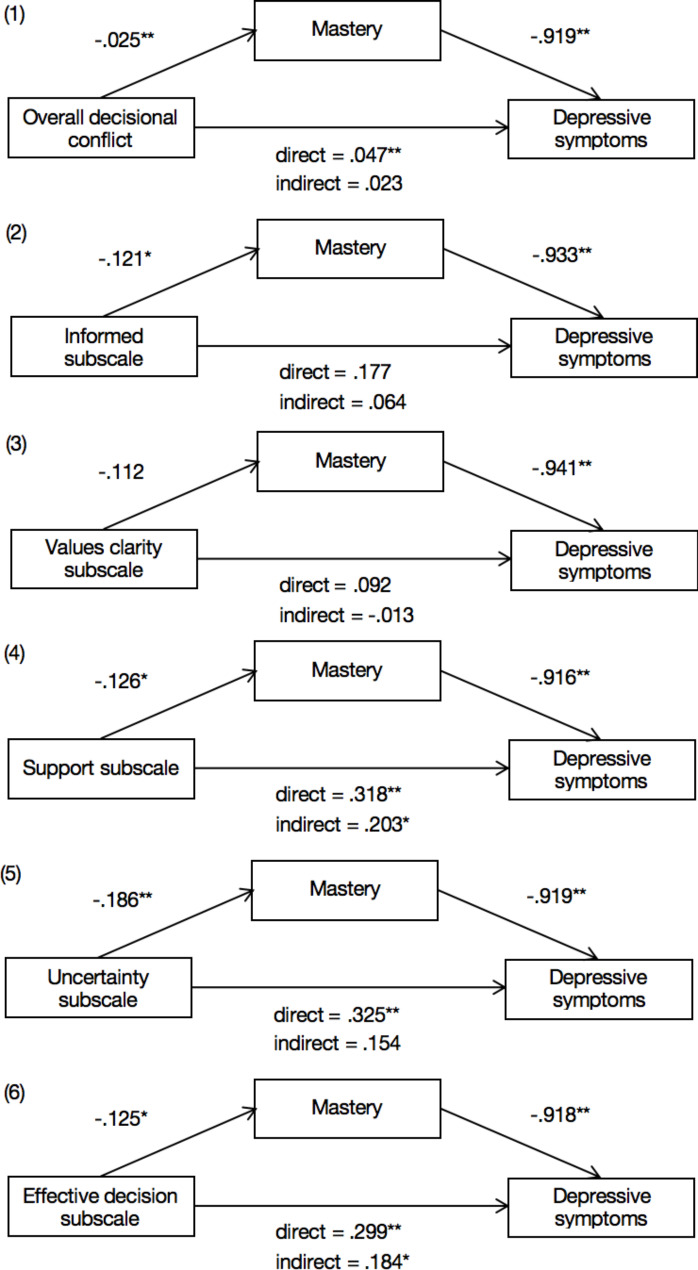
Simple mediation models for overall decisional conflict and the five dimensions of it the given numbers represent unstandardized coefficients. *p < 0.05, **p < 0.01

As shown in Table 3; Fig. 1, the total effects of overall effect of DC and three subscales on depressive symptoms were significant, except for decisional-making informed subscale (B = 0.177, SE = 0.112) and values clarity subscale (B = 0.092, SE = 0.119), and there were indirect effects of DC on depressive symptoms via mastery. Mastery demonstrated mediating effect, that is, the indirect effect of overall DC and other three subscales on depressive symptoms through mastery (mediator) were statistically significant (Overall DC: B = 0.023, SE = 0.009, 95% CI [0.008, 0.041]; decision support subscale: B = 0.115, SE = 0.054, 95% CI [0.015, 0.219]; decision-making uncertainty subscale: B = 0.171, SE = 0.063, 95% CI [0.048, 0.288]; effective decision-making subscale: B = 0.115, SE = 0.054, 95% CI [0.015, 0.206]). The direct effects of overall DC (B = 0.023, SE = 0.015) and decision-making uncertainty subscale (B = 0.154, SE = 0.105) were fully mediated by mastery. Among the three subscales, decision-making uncertainty subscale had the highest mediation effect.

**Table 3:** Mediation model testing the direct and indirect effects of overall decisional conflict on depressive symptoms via mastery

	Path a		Path b	Path a*b		Path c’	Path c	
Independent variable	Coef (BootSE)	R^2^	Coef (BootSE)	Coef (BootSE)	95% CI	Coef (BootSE)	Coef (BootSE)	R^2^ of mediation model
Overall decisional conflict	-0.025** (0.009)	0.056	-0.919** (0.090)	0.023* (0.009)	0.008–0.041	0.023 (0.015)	0.047** (0.017)	0.291
Informed subscale	-0.121*(0.061)	0.037	-0.933**(0.089)	0.113(0.058)	-0.001-0.236	0.064(0.098)	0.177(0.112)	0.287
Values clarity subscale	-0.112(0.065)	0.044	-0.941**(0.089)	0.105(0.062)	-0.001-0.221	-0.013(0.103)	0.092(0.119)	0.286
Support subscale	-0.126* (0.058)	0.057	-0.916** (0.089)	0.115* (0.054)	0.015–0.219	0.203* (0.092)	0.318** (0.106)	0.297
Uncertainty subscale	-0.186** (0.066)	0.059	-0.919** (0.090)	0.171** (0.063)	0.048–0.288	0.154 (0.105)	0.325** (0.120)	0.291
Effective decision subscale	-0.125* (0.058)	0.049	-0.918** (0.089)	0.115* (0.054)	0.015–0.206	0.184* (0.091)	0.299** (0.105)	0.295

Note: All coefficients (a, b, c’, c) were unstandardized coefficients

*p < 0.05, **p < 0.01

## Discussion

Results of the current study demonstrate a significant relationship between overall DC and depression in caregivers. Further, this study uncovered the mediating role of mastery between DC and depression. To be specific, mastery partially mediated the effect of effective decision-making and decision support on depression, and fully mediated the effect of overall DC and decision-making uncertainty on depression. In contrast, informed decision-making and values clarity were not significantly related to depression. Findings of the study can help researchers and healthcare professionals better understand how DC could affect depression, and can inform intervention strategies to enhance the psycho-social support to caregivers.

The original validation work reported that DCS had three subscales: decision-making uncertainty, factors contributing to the uncertainty, and effective decision-making. Later work further divided contributing factors into three subscales: informed decision-making, values clarity, and decision support [32]. The acquisition of information on treatment options, along with their benefits and risks, was believed to help resolve some issues caregivers may come across in treatment. However, contrary to what previous research suggest, our results showed that informed decision-making was not significantly related to mastery or depression. That indicates possible factors that may affect information receivers’ developing a sense of mastery. First, if treatment related information is presented to caregivers in a way making them aware of the information gaps to fill, and meanwhile motivating them to learn more, caregivers would experience a stronger sense of mastery. If not, the effect of the newly acquired information could be opposite, and learners could be discouraged from getting access to additional knowledge or developing a higher-level mastery [33, 34]. Second, acquisition of information about treatment does not necessarily mean the ability of effective processing and using information to keep healthy [35]. In the current information society, caregivers have access to information from multiple sources. They are likely to receive health-related information at such a high rate that it might be difficult for them to process effectively, and it is likely the information overload may reduce caregivers’ sense of mastery [36]. Future research should gain more insight into medical information processing and management that happen on parental caregivers.

Despite the insignificant effects from informed decision-making and values clarity, decision support, decision-making uncertainty, and effective decision-making were found significantly correlated with depression. Notably, in terms of uncertainty, many previous studies described it as a situation where caregivers held ambiguities about disease symptoms, health outcomes, and treatment [37–40]. Greater illness uncertainty was also found to be linked to greater depressive symptoms, and it has been targeted in clinical interventions that aimed to teach caregivers how to manage uncertainty to reduce distress [41, 42].

When interpreting the insignificant results on value clarification and significant results on decision support, the complex present situations of families’ decision-making and doctor-patient communication in China should be considered. First, deeply influenced by the collectivistic value, Chinese families highlight reciprocal relationships, and it is common that the extended family members (e.g., paternal and maternal grandparents) in China would participate in decision-making process throughout treatment phases. Therefore, treatment decision could be made under the dynamics of family power relations, rather than totally based on parental caregivers’ personal values or preference, even if parental caregivers play as the primary caregivers and make final decisions for children with illness [43, 44]. In other words, the potential inconsistency between family members’ opinions in decision-making could have a dysfunctional impact on parental caregivers’ decision-making effectiveness and their psychological well-being. Second, in China, doctors usually spend very limited time interacting with each outpatient or family caregiver due to the large number of outpatients. Due to that, discussion and communication with doctors often have to be medical problem focused. Previous studies also emphasized the power imbalance between doctors and patients. There is a lack of shared decision-making process to allow for sufficient discussion between family caregivers and doctors before they can fully take care of the values of each other and reach a satisfying agreement [45, 46]. Consequently, in this context, an individual’s acquisition of information and clarification of personal values is not enough to remove the barriers in decision making. Additionally, various needs could exist in oncological settings, so communication issues can be linked to unmet supportive care needs of patients and caregivers [47, 48]. In one word, simultaneous environmental support and care about caregivers’ decision-making system are indispensable for caregivers to develop the sense of control and have their mental well-being improved.

Prior studies have shown the association between mastery and caregiver outcomes, including physical health and depressive symptoms, as well as mastery’s mediating effect on the relationship between primary stressors and caregivers’ depressive symptoms [14, 49]. Interventions have been designed and implemented to improve caregivers’ mastery by improving their knowledge on illness, enhancing their problem-solving techniques, and teaching them relaxation skills [50, 51]. Although further investigation is needed, this current study suggests that support from healthcare workers and social workers would be valued in alleviating the uncertainty in making treatment decisions. Moreover, future investigations and efforts are needed in terms of supporting parental caregivers to get access to sufficient support and improving their sense of control.

### Clinical implications

The findings demonstrate the importance of enhancing caregivers’ mastery and resolving DC issues. Previous research have suggested interventions that focused on enhancing cognitive appraisal of caregiving (e.g., seeking positive aspects of caregiving, obtaining a sense of optimism related to care responsibilities) and providing health education to meet information needs are beneficial for caregivers to improve their sense of mastery [52, 53]. More than that, this study contributes to the existing knowledge by highlighting that sufficient support to address caregivers’ uncertainty in decision making process could even be more helpful than only providing caregivers with information related to medical treatment options. Recent Chinese studies advocated for improving doctor-patient communication [46] and developing caregivers’ coping strategies such as problem-focused (e.g., seeking help from supporting groups) and emotion-focused strategies (e.g., taking up sports, music, meditation as a way of distraction and self-enrichment) [54]. For healthcare staff in oncological settings, we suggested that a switch of efforts should be made from focusing on the efficiency to improving the quality of care and communication. Rather than just depending on doctors to achieve this goal, it would be more applicable with the involvement of medical social workers (e.g., facilitate family members to clarify their values, address parental and family conflicts that delay the consensus, provide emotional support) and the application of remote communication technique [55, 56]. The ongoing healthcare system reform in China should pay more attention to the community-level assistance to families of children with leukemia.

### Study limitations

When interpreting and applying the results, several limitations need recognition. First, this study only measured internal locus of control (mastery), since it had a stronger association with depression suggested by previous research. Future research is needed to examine and assess external locus of control (e.g., whether lean on powerful others, believe in fate, only accept recommendations of oncologists or not), as it might play an important role, specifically in mediating the effect of uncertainty, support, and effective decision [57]. Second, the sample was heterogeneous in terms of the types and severity of the illness, the time since diagnosis, and the phase of treatment. So the DC total score and subscale scores can fluctuate depending on the development of the illness and the timing of the assessment [58]. Additional research will be helpful to explore and compare DC in different phases of treatment with a larger sample size. Third, this study did not include grandparents and other informal caregivers of children, while they may actually take the primary caregiving responsibilities in many cases. Moreover, the current study did not have information on respondents’ evaluation about their interaction with doctors and other family members about treatment options. Future research should explore the effect of family involvement in decision making, and develop services to address DC in family decision making. Last, although the hypothesized models used in this study were based on SPM framework, the cross-sectional design of this study did not allow for establishing a causal relationship between variables. Future longitudinal studies are suggested to draw causal inferences.

## Conclusion

In conclusion, our study analyzed the relevance of DC, mastery, and depressive symptoms among parental caregivers of children with leukemia. It was found that an increased DC was associated with more depressive symptoms and lower sense of mastery. Among the five domains of DC, informed decision-making and values clarity were not significantly related to mastery or depression. Decision support, decision-making uncertainty, and effective decision-making were significantly related to depression, and their effects were partially or fully mediated by mastery. It is necessary to develop interventions focusing on social support to parental caregivers in decision-making process to improve their sense of mastery and psychological well-being.

## Data Availability

The datasets used and/or analysed during the current study available from the corresponding author on reasonable request.

## References

[1] Siegel RL, Miller KD, Jemal A (2019). Cancer statistics, 2019. Cancer J Clin.

[2] Lin X, Wang J, Huang X, Wang H, Li F, Ye W, Huang S, Pan J, Ling Q, Wei W (2021). Global, regional, and national burdens of leukemia from 1990 to 2017: a systematic analysis of the global burden of disease 2017 study. Aging.

[3] [http://www.nhc.gov.cn/wjw/zccl/201810/c90c2b2698ad40d2b1f1aa35ff991aa0.shtml].

[4] Al-Maliki SK, Al-Asadi J, Al-Waely A, Agha S (2016). Prevalence and levels of depression among parents of children with cancer in Basrah, Iraq. Sultan Qaboos University Medical Journal.

[5] Lovell B, Wetherell MA (2011). The cost of caregiving: endocrine and immune implications in elderly and non elderly caregivers. Neurosci Biobehavioral Reviews.

[6] Miller JJ, Morris P, Files DC, Gower E, Young M (2016). Decision conflict and regret among surrogate decision makers in the medical intensive care unit. J Crit Care.

[7] Beach SR, Schulz R, Yee JL, Jackson S (2000). Negative and positive health effects of caring for a disabled spouse: longitudinal findings from the caregiver health effects study. Psychol Aging.

[8] Loiselle M-C, Michaud C, O’Connor AM (2016). Decisional needs assessment to help patients with advanced chronic kidney disease make better dialysis choices. Nephrol Nurs J.

[9] Rahmani A, Azadi A, Pakpour V, Faghani S, Afsari EA (2018). Anxiety and depression: a cross-sectional survey among parents of children with cancer. Indian J Palliat Care.

[10] Rodriguez EM, Dunn MJ, Zuckerman T, Vannatta K, Gerhardt CA, Compas BE (2012). Cancer-related sources of stress for children with cancer and their parents. J Pediatr Psychol.

[11] Smeets SM, Van Heugten CM, Geboers JF, Visser-Meily JM, Schepers VP (2012). Respite care after acquired brain injury: the well-being of caregivers and patients. Arch Phys Med Rehabil.

[12] Pearlin LI, Bierman A. Current issues and future directions in research into the stress process. Handbook of the sociology of mental health. edn.: Springer; 2013: 325–40.

[13] Goldzweig G, Hasson-Ohayon I, Alon S, Shalit E (2016). Perceived threat and depression among patients with cancer: the moderating role of health locus of control. Psychol Health Med.

[14] Sherwood PR, Given BA, Given CW, Schiffman RF, Murman DL, Von Eye A, Lovely M, Rogers LR, Remer S (2007). The influence of caregiver mastery on depressive symptoms. J Nurs Scholarsh.

[15] Nijboer C, Tempelaar R, Triemstra M, van den Bos GA, Sanderman R (2001). The role of social and psychologic resources in caregiving of cancer patients. Cancer.

[16] Mausbach BT, Roepke SK, Chattillion EA, Harmell AL, Moore R, Romero-Moreno R, Bowie CR, Grant I (2012). Multiple mediators of the relations between caregiving stress and depressive symptoms. Aging Ment Health.

[17] Chan E-Y, Glass G, Chua K-C, Ali N, Lim W-S (2018). Relationship between mastery and caregiving competence in protecting against burden, anxiety and depression among caregivers of frail older adults. J Nutr Health Aging.

[18] Segrin C, Badger TA, Sikorskii A, Crane TE, Pace TW (2018). A dyadic analysis of stress processes in Latinas with breast cancer and their family caregivers. Psycho-oncology.

[19] Ajay S, Kasthuri A, Kiran P, Malhotra R (2017). Association of impairments of older persons with caregiver burden among family caregivers: findings from rural South India. Arch Gerontol Geriatr.

[20] Pearlin LI, Mullan JT, Semple SJ, Skaff MM (1990). Caregiving and the stress process: an overview of concepts and their measures. Gerontologist.

[21] Ryff CD (1989). Happiness is everything, or is it? Explorations on the meaning of psychological well-being. J Personal Soc Psychol.

[22] O’Connor AM, Tugwell P, Wells GA, Elmslie T, Jolly E, Hollingworth G, McPherson R, Bunn H, Graham I, Drake E (1998). A decision aid for women considering hormone therapy after menopause: decision support framework and evaluation. Patient Educ Couns.

[23] Vanderhorst RK, McLaren S (2005). Social relationships as predictors of depression and suicidal ideation in older adults. Aging Ment Health.

[24] Chiavari L, Gandini S, Feroce I, Guerrieri-Gonzaga A, Russell-Edu W, Bonanni B, Peccatori FA (2015). Difficult choices for young patients with cancer: the supportive role of decisional counseling. Support Care Cancer.

[25] Guadagno MA. Health decisions for others: an extension of the Health Belief Model. 2017.

[26] Ssegonja R, Sampaio F, Alaie I, Philipson A, Hagberg L, Murray K, Sarkadi A, Langenskiöld S, Jonsson U, Feldman I (2020). Cost-effectiveness of an indicated preventive intervention for depression in adolescents: a model to support decision making. J Affect Disord.

[27] LeBlanc TW, Bloom N, Wolf SP, Lowman SG, Pollak KI, Steinhauser KE, Ariely D, Tulsky JA (2018). Triadic treatment decision-making in advanced cancer: a pilot study of the roles and perceptions of patients, caregivers, and oncologists. Support Care Cancer.

[28] Gower WA, Golden SL, King NM, Nageswaran S (2020). Decision-making about tracheostomy for children with medical complexity: Caregiver and health care provider perspectives. Acad Pediatr.

[29] Andresen EM, Malmgren JA, Carter WB, Patrick DL (1994). Screening for depression in well older adults: evaluation of a short form of the CES-D. Am J Prev Med.

[30] O’Connor AM (1995). Validation of a decisional conflict scale. Med Decis Making.

[31] Pearlin LI, Schooler C. The structure of coping. J Health Soc Behav 1978:2–21. 649936

[32] Lam WW, Kwok M, Liao Q, Chan M, Or A, Kwong A, Suen D, Fielding R (2015). Psychometric assessment of the C hinese version of the decisional conflict scale in C hinese women making decision for breast cancer surgery. Health Expect.

[33] Murayama K, FitzGibbon L, Sakaki M (2019). Process account of curiosity and interest: a reward-learning perspective. Educational Psychol Rev.

[34] Camfield EK, Beaster-Jones L, Miller AD, Land KM. Using writing in science class to understand and activate student engagement and self-efficacy. Active learning in College science. edn.: Springer; 2020: 89–105.

[35] Liu C, Wang D, Liu C, Jiang J, Wang X, Chen H, Ju X, Zhang X. What is the meaning of health literacy? A systematic review and qualitative synthesis. Family Med community health 2020, 8(2). 10.1136/fmch-2020-000351 PMC7239702 32414834

[36] Kim S (2021). Caregivers’ information overload and their personal health literacy. West J Nurs Res.

[37] Mishel MH (1984). Perceived uncertainty and stress in illness. Res Nurs Health.

[38] Mishel MH (1990). Reconceptualization of the uncertainty in illness theory. Image: The Journal of Nursing Scholarship.

[39] Byun E, Riegel B, Sommers M, Tkacs N, Evans L (2017). Effects of uncertainty on perceived and physiological stress in caregivers of stroke survivors: a 6-week longitudinal study. J Gerontol Nurs.

[40] Coppock C, Ferguson S, Green A, Winter D (2018). It’s nothing you could ever prepare anyone for’: the experiences of young people and their families following parental stroke. Brain Injury.

[41] Mullins LL, Cushing CC, Suorsa KI, Tackett AP, Molzon ES, Mayes S, McNall-Knapp R, Mullins AJ, Gamwell KL, Chaney JM (2016). Parent illness appraisals, parent adjustment, and parent-reported child quality of life in pediatric cancer. Pediatr Hematol Oncol.

[42] Mullins LL, Fedele DA, Chaffin M, Hullmann SE, Kenner C, Eddington AR, Phipps S, McNall-Knapp RY (2012). A clinic-based interdisciplinary intervention for mothers of children newly diagnosed with cancer: a pilot study. J Pediatr Psychol.

[43] Zhai H, Lavender C, Li C, Wu H, Gong N, Cheng Y (2020). Who decides? Shared decision-making among colorectal cancer surgery patients in China. Support Care Cancer.

[44] Huang Y, Cong Y. Ethical Consideration about Family Members’ Participation in Cancer Treatment Decision-making. Chin Med Ethics 2017:315–8.

[45] Sun C, Zou J, Zhao L, Wang Q, Zhang S, Ulain Q, Song Q, Li Q (2020). New doctor-patient communication learning software to help interns succeed in communication skills. BMC Med Educ.

[46] Tu J, Kang G, Zhong J, Cheng Y (2019). Outpatient communication patterns in a cancer hospital in China: a qualitative study of doctor–patient encounters. Health Expect.

[47] Maguire P, Faulkner A (1988). Communicate with cancer patients: 1. Handling bad news and difficult questions. BMJ: Br Med J.

[48] de Heus E, van der Zwan JM, Husson O, Frissen AR, van Herpen CM, Merkx MA, Duijts SF (2021). Unmet supportive care needs of patients with rare cancer: a systematic review. Eur J Cancer Care.

[49] Yeh P-M, Wierenga ME, Yuan S-C (2009). Influences of psychological well-being, quality of caregiver-patient relationship, and family support on the health of family caregivers for cancer patients in Taiwan. Asian Nurs Res.

[50] Chiu M, Wesson V, Sadavoy J (2013). Improving caregiving competence, stress coping, and mental well-being in informal dementia carers. World J Psychiatry.

[51] Schwarz KA, Mion LC, Hudock D, Litman G (2008). Telemonitoring of heart failure patients and their caregivers: a pilot randomized controlled trial. Prog Cardiovasc Nurs.

[52] Gaugler JE, Linder J, Given CW, Kataria R, Tucker G, Regine WF (2009). Family cancer caregiving and negative outcomes: the direct and mediational effects of psychosocial resources. J Fam Nurs.

[53] Wang J, Yao N, Shen M, Zhang X, Wang Y, Liu Y, Geng Z, Yuan C (2016). Supporting caregivers of children with Acute Lymphoblastic Leukemia via a smartphone app: a pilot study of usability and effectiveness. Comput Inf Nurs.

[54] Yeung NCY, Cheung KC, Chau HC, Leung AWK, Li CK, Lam TTN, Cheng HY, Cheung YT. Transition from Acute Treatment to Survivorship: exploring the psychosocial adjustments of chinese parents of children with Cancer or Hematological Disorders. Int J Environ Res Public Health 2021, 18(15). 10.3390/ijerph18157815 PMC8345777 34360108

[55] Tu J, Wang C, Wu S (2018). Using technological innovation to improve health care utilization in China’s hospitals: the emerging ‘online’health service delivery. J Asian Public Policy.

[56] Elwyn G, O’Connor A, Stacey D, Volk R, Edwards A, Coulter A, Thomson R, Barratt A, Barry M, Bernstein S (2006). Developing a quality criteria framework for patient decision aids: online international Delphi consensus process. BMJ.

[57] Dopelt K, Bashkin O, Asna N, Davidovitch N (2022). Health locus of control in cancer patient and oncologist decision-making: an exploratory qualitative study. PLoS ONE.

[58] Garvelink MM, Boland L, Klein K, Nguyen DV, Menear M, Bekker HL, Eden KB, LeBlanc A, O’Connor AM, Stacey D (2019). Decisional conflict scale use over 20 years: the anniversary review. Med Decis Making.

