# Correction: Decisional conflict, caregiver mastery, and depression among Chinese parental caregivers of children with leukemia

**DOI:** 10.1186/s12888-023-05255-0

**Published:** 2023-10-11

**Authors:** Mowen Liu, Weizhou Tang, Ye Zhang, Wenjun Sun, Yang Wang

**Affiliations:** 1 Department of Social Work and Social Policy, Renmin University of China, Beijing, China; 2 Beijing Yizhuang Technology Innovation Company Limited, Beijing, China; 3 Charles R. Drew University of Medicine and Science, Los Angeles, United States; 4 the High School Affiliated to Renmin University of China, Beijing, China; 5 Tianjin Di Ai Zhi Jia Hard-pressed Families Service Center, Tianjin, China

**Correction: BMC Psychiatry (2023) 23:625**

10.1186/s12888-023-05084-1

Following the publication of the original article [1], the authors identified errors in the Methods section. The symbol XX has been replaced with Renmin University of China.

The original article [1] has been corrected.
